# An Overview of Multimodal Neuroimaging Using Nanoprobes

**DOI:** 10.3390/ijms18020311

**Published:** 2017-02-01

**Authors:** Sriram Sridhar, Sachin Mishra, Miklós Gulyás, Parasuraman Padmanabhan, Balázs Gulyás

**Affiliations:** 1 School of Electrical & Electronic Engineering, Nanyang Technological University, 50 Nanyang Avenue, Singapore 639798, Singapore; sriram005@e.ntu.edu.sg; 2 Lee Kong Chian School of Medicine, Nanyang Technological University, 59 Nanyang Drive, Singapore 636921, Singapore; sachin.mishra@ntu.edu.sg; 3 Department of Immunology, Genetics and Pathology, Rudbeck Laboratory, Uppsala University, Dag Hammarskölds väg 20, Uppsala Se-751 85, Sweden; miklos.gulyas@igp.uu.se

**Keywords:** nanoprobes, multimodal imaging, neuroimaging, tumor

## Abstract

Nanomaterials have gained tremendous significance as contrast agents for both anatomical and functional preclinical bio-imaging. Contrary to conventional medical practices, molecular imaging plays an important role in exploring the affected cells, thus providing precision medical solutions. It has been observed that incorporating nanoprobes improves the overall efficacy of the diagnosis and treatment processes. These nano-agents and tracers are therefore often incorporated into preclinical therapeutic and diagnostic applications. Multimodal imaging approaches are well equipped with nanoprobes to explore neurological disorders, as they can display more than one type of characteristic in molecular imaging. Multimodal imaging systems are explored by researchers as they can provide both anatomical and functional details of tumors and affected tissues. In this review, we present the state-of-the-art research concerning multimodal imaging systems and nanoprobes for neuroimaging applications.

## 1. Introduction

The success of medicine and healthcare is largely determined by the efficacy of treatment techniques. By incorporating functionalized nanoprobes and/or tracers with imaging systems, better results can be achieved for diagnosis and treatment of neurological diseases [1,2]. Specifically, the introduction of a targeted molecular agent therapy may be accompanied by feedback schemes that allow for real-time monitoring of the disease and agent reaction patterns, which can help achieve the objective of drug optimization and personalized healthcare [3,4,5].

Imaging systems serve as an excellent diagnostic tool by capturing the details of the disease and therapeutic process on injecting bio-chemical agents or nanoprobes. Several imaging techniques have been developed over time to study the effects of therapeutic agents [6]. In many cases, two or more of such imaging systems are chosen to simultaneously acquire and examine the target area. In other cases, images may be captured at different time intervals using different modalities within the asynchronous model of acquisition for which effective post-processing solutions should be developed. This marriage between therapeutics and diagnostics, as well as the choice of imaging systems, is greatly influenced by the design of the nano-imaging agents. Multimodal systems in general allow for utilizing the strengths of each imaging system while overcoming their limitations.

The most frequently used imaging systems in diagnostic applications include Positron Emission Tomography (PET), Computed Tomography (CT), Single-Photon Emission Computed Tomography (SPECT) and Magnetic Resonance Imaging (MRI). By definition, depending on the requirement, these modalities are appropriately chosen in specific combinations. Histed et al. [7] explains the need for hybrid imaging in the context of capturing the anatomical and functional details of a target by providing concrete examples for current and future use. Ripen et al. [8] presents the idea of multimodal imaging and some of the latest trends and developments in this area. Marti-Bonmati et al. [9] presents the most commonly used multimodal techniques in diagnostic imaging while also outlining the new ones in the field. Cherry et al. [10] explores the multimodal imaging systems that can be used for medical applications beyond hybrid combinations that have been used conventionally.

As previously mentioned, the role of theranostic agents is extremely important and cannot be ignored. In addition to fulfilling therapeutic requirements, they must also yield themselves to imaging and diagnostic requirements. Iron oxide nanoparticles [11,12], gold [13], carbon dots, etc., have been typically demonstrated to be applicable for various preclinical theranostic applications. Xie et al. [14] explains the research in the direction of nano-platforms which serve the dual purpose of ferrying drugs to the site in the body as well as exhibiting imaging characteristics. Simon et al. [15] present the application of gold-based nano-aggregates coated with methylene blue for multimodal imaging applications. Rai et al. [16] presents a class of photo-triggered theranostic agents by reviewing the developments in their usage for photo-dynamic, photo-thermal, and photo-triggered chemotherapy for diseases.

Owing to the significant advances in nanoprobes, multimodal imaging and the plethora of works available in the field, there is an impending need to bring to order the latest trends, which is the focus of this paper. In this review, we classify the existing literature according to multimodal imaging system used. In doing so, we provide a comprehensive overview of the nanoprobes/tracers used for neuroimaging applications and the context in which they are used, classified as per the multimodal combinations as PET-CT, PET-MRI and SPECT-CT.

This paper is subsequently organized as follows. Section 2 describes the most recent studies underway in multimodal imaging enhanced with nanoprobes under the domain of PET-CT, PET-MRI and SPECT-CT for neuroimaging applications. Section 3 presents the conclusion of this review.

## 2. Multimodal Imaging with Nanoprobes

### 2.1. Imaging with Positron Emission Tomography/Computed Tomography (PET-CT)

In this section, we discuss the works employing PET-CT as the imaging modality for neurological applications with nanoprobes as contrast agent. The need for this integrated combination is directly related to the desire to overcome the shortcomings of using only one modality or multiple modalities independently. In this case, PET has an innate ability to determine the metabolic activity of tissues but cannot provide details at a high resolution. Although CT cannot provide for sharp differences in physiology, it is the perfect companion to the PET for high resolution anatomical details. This potent combination is therefore a good solution for imaging and lesion localization [17].

**Figure 1:**
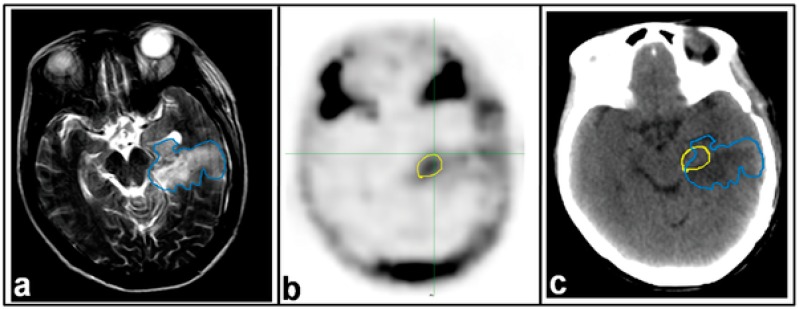
Magnetic Resonance Imaging (MRI) and ^18^F–Fluro-ethyl-tyrosine (^18^F–FLT)/Positron Emission Tomography/Computed Tomography (PET-CT) images from a patient with glioblastoma (GBM). Images were taken 21 days post-operatively and 2 days before radiotherapy. (**a**) T2-weighted MRI; (**b**) ^18^F–FLT PET image; (**c**) CT image of PET-CT scan. Residual tumor regions defined by T2-MRI (blue line) and ^18^F–FLT PET (yellow line) are superimposed on the CT image. This research was originally published in Reference [18].

Zhao et al. [18] compares the presence of residual disease post operation in patients with malignant glioma using MRI and PET-CT imaging. Flurothymidine (^18^F–FLT) was used as the radiotracer for imaging using PET-CT as shown in Figure 1. Furthermore, the impact of ^18^F–FLT PET on the estimation of post-operative target volumes for radiotherapy was investigated. The authors concluded that post-surgery tumor volumes detected by ^18^F–PET are not always consistent with those obtained through MRI and that incorporation of ^18^F–FLT-PET significantly improves the delineation of target volume for radiotherapy.

Badakhshi et al. [19] presents the application of ^18^F–Fluro-ethyl-tyrosine (^18^F–FET) to identify the total tumor volume in skull-base lesions for image-guided stereotactic radiotherapy with PET-CT. They also show the comparative advantages of the FET tracer for target identification in the therapy planning process. Unlike FDG-PET, better accuracy detection rates may be obtained by employing FET-PET. ^18^F–FET demonstrates a much lower sensitivity but a higher specificity compared to FDG. Furthermore, ^18^F–FET allows a precise estimation of cranial border tumors compared to FDG.

Nonokuma et al. [20] presents the study of regional cerebral glucose metabolism in patients who are suffering from malignant lymphoma using FDG-PET-CT imaging and analysis thereupon. Under their proposed approach, whole-body scans (including brain) were taken and the regional cerebral glucose mechanism on a voxel by voxel was estimated using statistical parametric mapping. Based on their experiments, the authors also reported that patients with malignant lymphoma exhibit severe cerebral glucose metabolism dysfunction.

Deguchi et al. [21] presented a case study of the development of cerebral and cerebellar lesions following the coiling procedure using PET-CT as the hybrid modalities in addition to the MRI and proton magnetic resonance spectroscopy (H-MRS). ^11^C–Methionine (MET-PET) was used as the tracer for experiment using PET-CT. This is because the uptake of Methionine corresponds to the presence of lesions in the brain following the coiling procedure. The authors highlight the importance of conducting such a diagnostic imaging following coiling failing as the lesions could go unnoticed.

Wright et al. [22] propose a model to determine the infarction cerebral blood flow (CBF) thresholds at three-hour ischemia time by comparing CBF maps arising from CT Perfusion to ^18^F–FFMZ-PET images. As can be seen, the tracer agent used in this work is fluoroflumazenil because it can be reliably used to predict the final infarct with relatively few false positives. Zhang et al. [23] highlighted the differences in glucose metabolism in the brain between patients with non-small cell lung cancer and control participants using PET-CT imaging with FDG as the tracer. FDG tracer is used as it can be used to analyze the tissue metabolic activity by virtue of the glucose intake in the region. It was observed that, in using this tracer, there was a difference in the FDG metabolism computed as normalized signal intensity ratio of each brain region to that of the whole brain between lung cancer patients and the control group using statistical tests. The authors inferred that the changes in blood glucose metabolism in subjects suffering from non-small cell lung cancer might be due to lung cancer-related visceral sympathetic activation and decrease of dorsal attention network function.

**Figure 2:**
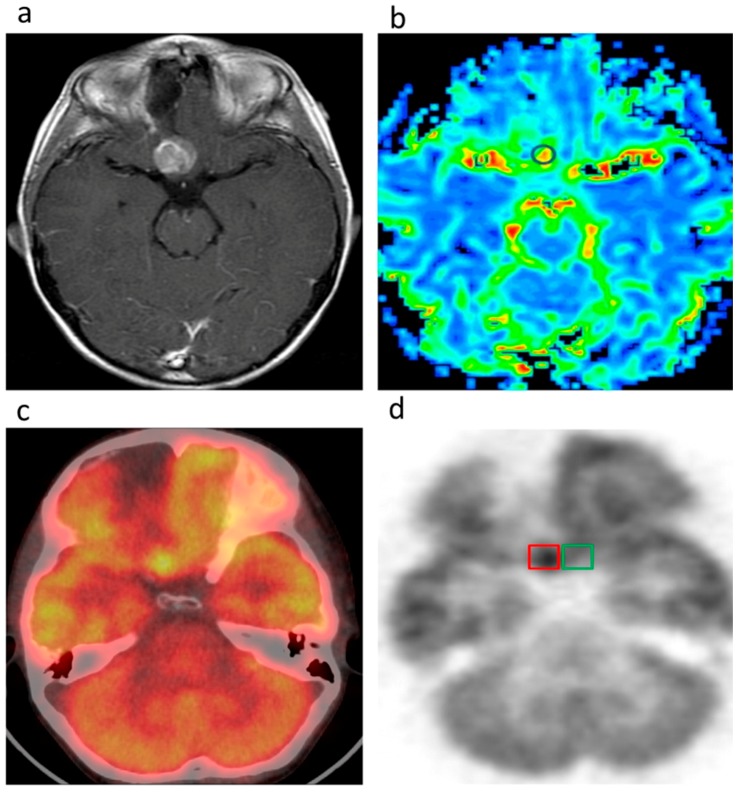
This male patient with a history of right frontal lobe GBM presents with increased enhancement on axial T1-weighted imaging (**a**) 12.3 months after completion of radiation therapy. The region of interest analyses for the MRI perfusion (**b**) and FDG PET-CT (**c**,**d**) examinations demonstrate an rCBV_max_ of 3.6 and SUV_ratio_ of 2.0. The SUV_lesion max_ is 8.2 (red box), and the SUV_normal brain_ is 4.1 (green box). The lesion was resected and pathologically proven to be recurrent tumor. Adapted with permission from Reference [25].

Knesaurek et al. [24] demonstrated the application of hybrid Fourier-wavelet windowed Fourier transform restoration technique to qualitatively enhance the images of Alzheimer’s disease acquired through the ^18^F–FDG-PET-CT system. Hatzoglou et al. [25] focused on differentiating radiation injury from brain tumor for optimal and personalized treatment. They utilize both MRI Perfusion as well as ^18^F–FDG-PET-CT, while also assessing the effectiveness of each in order to achieve the objective as shown Figure 2. Their studies indicate that both systems show similar performance in identifying tumor from radiation injury. They also conjecture that altering the SUV_ratio_ may improve the efficacy of FDG-PET-CT as the imaging modality for this application. Lapa et al. [26] investigated the expression of Chemokine receptor-4 in glioblastoma using the non-invasive ^68^Ga–Pentixafor-PET-CT imaging thereby allowing for the quantification of Chemokine receptor-4 (CXCR4) and the identification of suitable patients for therapy. ^68^Ga–Pentixafor-PET-CT was chosen as the imaging agent because of its strong affinity for CXCR4, which is reportedly overexpressed in glioblastoma lesions.

### 2.2. Imaging with PET-Magnetic Resonance Imaging (MRI)

As the name suggests, this hybrid imaging technique combines the power and flexibility of PET and MRI to avoid the pitfalls caused by using each modality independently. Jadvar et al. [27] describes the potential benefits of PET-MRI which, in certain clinical settings, override the benefits offered by PET-CT, thereby making it a useful choice for such applications. In the following paragraph, we list recent works employing PET-MRI for neuroimaging applications.

Guo et al. [28] examined the relationship between brain functions, aging and caloric restriction on young and old mice. They pay close attention to the glucose metabolism in the brain, energy metabolites and the integrity of white matter in mice of different age groups fed with either limited or 40% caloric restriction meals. Unlike normal aging caloric restriction, aging is characterized by preservation of energy metabolites, white matter integrity and long-term memory in old mice. The authors concluded that caloric restriction affects aging by slowing down the process. They also experimentally ascertained the age-dependent effects of caloric restriction through neuroimaging (PET/MRI/MRS) techniques.

**Figure 3:**
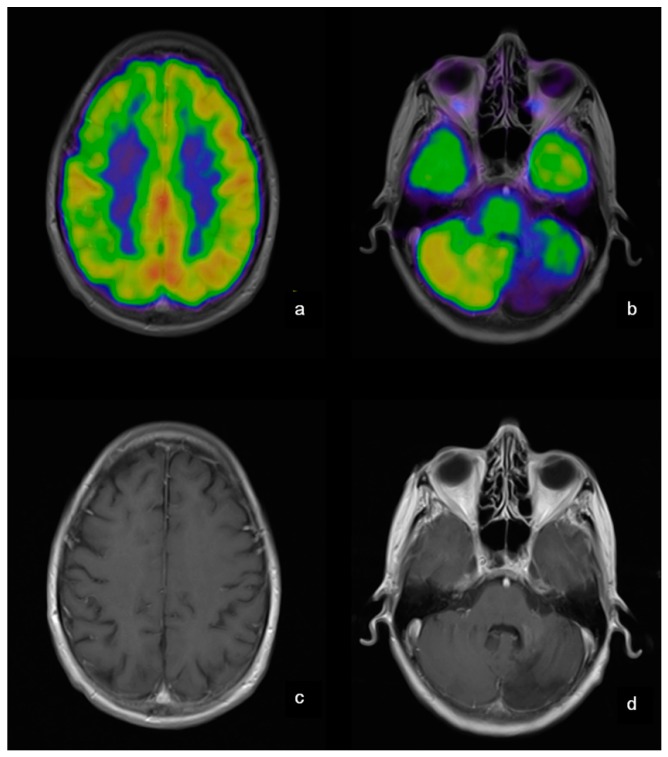
Patient no. 25 suffered from a cerebellar hemorrhage and developed a hypometabolic area corresponding to tissue loss in the left cerebellar hemisphere (b + d) in addition to a cerebellar cortical diaschisis (a + b). (a) Cortex (MRI fusioned with PET)–decreased activity in the right hemisphere; (b) Cerebellum (MRI fusioned with PET)—decreased activity in the left cerebellar hemisphere; (c) Cortex (MRI)—no structural changes; (d) Cerebellum (MRI)—sequelae after hemorrhage. This research was originally published in Reference [30].

Werner et al. [29] employed 15O-H_2_O as tracer for PET/ MRI on stroke patients to overcome the limitation of single modality (MRI) information in helping identify critically hypo-perfused tissue during thrombolysis trials. This augmentation of standard stroke MRI with PET data can pave the way for better patient stratification and personalized healthcare. As opposed to the sequential model, simultaneous data acquisition using 15O-H_2_O with PET/MRI was used to relate Cerebral Blood Flow (CBF) measurements of MRI data with that of PET data, although the basic stroke care itself remains unchanged. Korsholm et al. [30] assesses the brain activity and functions in Danish patients with Fabry disease using FDG-PET-MRI as shown in Figure 3. FDG was used as the nanoprobes to record the relative values of regional cerebral glucose metabolic rate in patients with Fabry disease. Surprisingly, based on the acquired data, the authors inferred that FDG-PET adds little value to the MRI data obtained in patients with Fabry disease, thereby making MRI the modality of choice when monitoring the cerebral status. Jena et al. [31] used FDG-PET-MRI modality to monitor dementia patients which is presented as a case study. Patients upon fasting were injected with the FDG tracer and were then subjected to PET-MRI examination. Signs of abnormality such as hypometabolism or any visual defects are then identified in close association with diagnosis and follow-up action.

**Figure 4:**
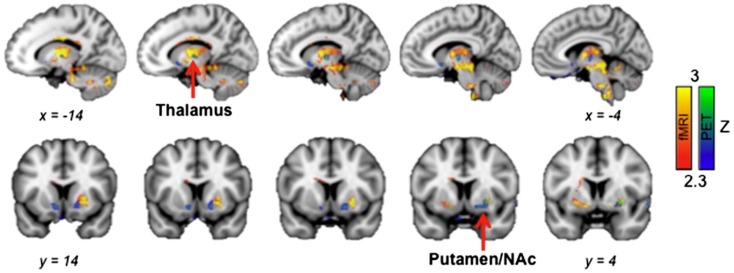
fMRI-PET activation overlaps in responses to external pain stimuli in a group of healthy volunteers. Spatial overlap of receptor activation measured decreases in BP_ND_ (PET, blue–green) and BOLD fMRI (pain > non-painful pressure, red–yellow) was shown in the thalamus and striatum (putamen/nucleus accumbens) (Putamen/NAc). Adapted with permission from Reference [33].

Anazodo et al. [32] discussed the approach of adding bone information to improve the attenuation correction of the PET in a hybrid PET-MRI whole-brain imaging system which can qualitatively improve the performance of the system. This information is otherwise missing on the Dixon attenuation correction. In this research, FDG was the nanoprobe used with PET imaging. The data acquired with this FDG-based imaging system was compared with CBF to investigate the potential clinical applications of their enhanced MRAC method. Hsiao Ying Wey et al. [33] discussed the opiodergic pain system in the human brain through simultaneous fMRI-PET image acquisition as shown in Figure 4. The opioid radioligand ^11^C diprenorphine (^11^C–DPN) was chosen as the radiotracer for their experiments to identify the regional endogenous opioid displacement from the opioid receptor. The objective of their work was to identify where in the pain-activated brain network opioid modulation occurs. Through their investigations, the authors establish the co-localized occurrence of responses in the thalamus and striatum-related pain processing. These signal changes in the thalamus were positively correlated indicating that the opioid neurotransmitter changes induced by pain make up a significant component of the fMRI signal changes.

Zandieh et al. [34] employed ^18^F–FDG-PET-MRI data to analyze the metabolic and structural changes in patients suffering from torture-related post-traumatic stress disorder. FDG tracer-based PET images are used to study metabolism in the different brain regions in the patients. They observed that the post-torture trauma was inimical to the morphology and the functions of the brain which was well captured through the use of the PET-MRI hybrid imaging system.

Lewis et al. [35] explored the usage of divalent metal transporter 1 (DTM1) reporter gene for cell tracking in the central nervous system. Specifically, they investigated Mn-based PET-MRI and established the proof of concept of DTM1 as a reporter gene working on stem cells in the rat brain. ^52^Mn was produced in their experiments. By virtue of overexpression of the protein, increased Mn incorporation can be used for selective signal enhancement using the MRI making them suitable for cellular imaging. Henriksen et al. [36] used ^18^F–FET-PET-DSC MRI for evaluating tumor metabolism, structure and blood volume in the study of the human brain. ^18^ F–FET is used as the nanoprobe for this application as it can be used to investigate the transport of amino acids, which is a valuable addition in the evaluation of patients with brain tumor.

### 2.3. Imaging with Single-Photon Emission Computed Tomography (SPECT)-CT

The SPECT-CT hybrid combination is yet another popularly used imaging system in medical theranostics because of its superior performance in lesion characterization, representation and findings as reported in [37,38,39]. Bural et al. [40] highlighted the comparative advantages offered by SPECT-CT over the unimodal SPECT system in the problem of localizing endocrine and neuroendocrine tumors, thereby also implicitly emphasizing the significance of the multimodal imaging system. Mariani et al. [41] reviews the clinical applications of SPECT-CT and its importance, even with the emergence of PET as an advanced alternative. There are very few research models that focus on human brain imaging with SPECT-CT and radiotracers.

Rangger et al. [42] used dedicated multipurpose liposomal nanoparticles not only for serving as therapy vehicles but also to lend themselves to multimetric imaging. SPECT-CT was the dominant imaging system used, although preliminary MRI studies were conducted. The developed theranostic agent displayed radioactive, fluorescent and magnetic resonance signaling properties.

Bäck et al. [43] employed ^123^I-β-CIT-SPECT-CT to evaluate the condition of the rat midbrain dopaminergic pathway in the developed 6-hydroxydopamine model of Parkinson’s disease. In order to synthesize a model of Parkinson’s disease, 6-Hydroxydopamine (6-OHDA) was used to induce the degeneration of neurons in rats. The dopamine transporter radio ligand ^123^I-β-CIT was used to estimate the DAT density in the striatum of the lesion-induced rats. The termination of dopamine signaling is the sole responsibility of DAT. As the DAT transporter is present only in the plasma membrane of the dopamine neurons in the central nervous system, it is an excellent biomarker for this network.

Pitkonen et al. [44] investigated the capturing and measuring capability of a high-resolution SPECT-CT system of the ^123^I-β-CIT dopamine transporter binding in the mouse brain which can prove useful for ascertaining neuroprotective properties of drugs in mice. Unlike previous works, which have employed ^123^I-β-CIT for imaging operations in rats and monkeys, this work strives to do so in mice. ^123^I-β-CIT exhibits good correlation with the Binding Potential (BP) which is directly proportional to the density of DAT in equilibrium. It is BP which is used to assess the ^123^I-β-CIT ratios in the studies of mice. Further, ^123^I-β-CIT has several advantages to offer, the main reason being the higher binding ratios being reported in humans thereby increasing the signal to noise ratio while operating and reporting from the data on the mouse brain. Their work is the first to report the 3D kinetics of ^123^I-β-CIT in mice.

In addition to these studies, functionalized nanoprobes are also developed for neuroimaging applications for other multimodal imaging systems such as PET-Fluorescence and MRI-Fluorescence. Table 1 lists the recently developed nanoprobes used for multimodal neuroimaging applications.

## 3. Discussion and Future Research Directions

Although the multimodal imaging systems and probes available today offer several benefits in the preclinical and clinical arenas, there are potential loopholes or scope for future research that may have to be taken into consideration. Literature [45,46] suggests that there is an impending need to develop new nano-agents that lend themselves to imaging using multiple modalities and many applications. Nano-agents such as FDG, although widely successful with unimodal imaging solutions such as PET, still suffer when used with multiple imaging systems such as PET-CT in applications with low FDG avidity, such as prostate cancer, for which other tracers have to be developed. An equally important direction for research is in looking out for alternatives in the multimodal systems themselves or new combinations of them [10]. For example, the pitfalls of CT as an anatomical imaging modality are well known and documented. Lastly, careful consideration should also be given to the process of imaging itself. As important as it is to get high-quality images, other aspects must be given due consideration, such as dosage, exposure to radiation, cost, image-acquisition rate, tolerance level of patients etc., which impact the overall diagnostic imaging process. These aforementioned factors may therefore guide the direction for future research in biomedical imaging.

## 4. Conclusions

This paper provided an overview of the latest developments in the field of multimodal neuroimaging using nanoprobes, in combination with functional and anatomical imaging. This provides an insight into various probes and tracers developed for preclinical imaging with multimodal approaches such as PET-CT (Positron Emission Tomography/Computed Tomography), PET-MRI (Magnetic Resonance Imaging), SPECT-CT (Single-Photon Emission Computed Tomography), etc. The applicability of these probes is well demonstrated through various aspects of functional imaging in brains through PET and SPECT, together with anatomical CT or MRI images. These applications include basic metabolism of brain, neurodegenerative diseases, lesions, tumors, aging, cognitive dysfunctions, drug effects, blood flow study and various others. Multimodal imaging in these applications allows for capturing different aspects of the brain process while also overcoming the shortcomings of unimodal systems, thus making them a popular choice. It can therefore be concluded that multimodal imaging systems with nanoprobes are well demonstrated in preclinical setup for neuroimaging applications and, together with the upcoming research models, the path for clinical diagnostic, therapeutic and theranostic applications is being paved.

## Figures and Tables

**Table 1:** Recently developed nanoprobes used for multimodal neuroimaging applications.

S. No.	Nanoprobes	Imaging Modalities	Application	References
1	Flurothymidine (^18^F–FLT)	MRI and PET-CT	Detecting residual disease post operation in patients with malignant glioma	Zhao et al. [18]
2	^18^F–Fluro-ethyl-tyrosine (^18^F–FET)	PET-CT	Image-guided stereotactic radiotherapy in patients with skull-base lesions	Badakhshi et al. [19]
3	FDG	PET-CT	Statistical parametric mapping, metabolic activities visualization	Nonokuma et al. [20]
4	^11^C–Methionine	PET-CT	Cerebellar lesions imaging	Deguchi et al. [21]
5	Fluoroflumazenil	PET-CT	Cerebral blood flow during heart attacks	Wright et al. [22]
6	^18^FDG	PET-CT	Modelling Alzheimer’s using Fourier transform analysis	Knesaurek et al. [24], Hatzoglou et al. [25]
7	^68^Ga–Pentixafor	PET-CT	Studying Chemokine receptor 4 during glioblastoma	Lapa et al. [26]
8	^18^FDG	PET/MRI/MRS	Determine relationship between brain functions, aging and caloric restriction	Guo et al. [28]
9	^15^O-H_2_O	PET/ MRI	To identify critically hypo perfused tissue during thrombolysis trials	Werner et al. [29]
10	^18^FDG	PET/MRI	Record the relative values of regional cerebral glucose metabolic rate in Fabry disease and monitoring dementia	Korsholm et al. [30], Jena et al. [31]
11	^18^FDG	PET/MRI	Whole-brain imaging system	Anazodo et al. [32]
12	^11^C diprenorphine (^11^C–DPN)	fMRI/PET	To identify the regional endogenous opioid displacement from the opioid receptor	Hsiao Ying Wey et al. [33]
13	^18^FDG	PET/MRI	Study metabolism in the different brain regions	Zandieh et al. [34]
14	^52^Mn	PET/MRI	Divalent metal transporter 1 (DTM1) reporter gene for cell tracking in the central nervous system	Lewis et al. [35]
15	^18^F–FET	PET-DSC MRI	Evaluating tumor metabolism, structure and blood volume in the study of the human brain	Henriksen et al. [36]
16	Liposomal nanoparticles	SPECT-CT	Localizing endocrine and neuroendocrine tumors, brain imaging	Pachowicz et al. [37], Ndlovu et al. [38], Helyar et al. [39], Rangger et al. [42]
17	^123^I-β-CIT	SPECT-CT	Synthesized a model of Parkinson’s using 6-Hydroxydopamine (6-OHDA) pathway in rats, for ascertaining neuroprotective properties of drugs	Bäck et al. [43]
18	^123^I-β-CIT	SPECT-CT	Dopamine transporter binding in the mouse brain	Pitkonen et al. [44]
